# Decreased Expression of ACADSB Predicts Poor Prognosis in Clear Cell Renal Cell Carcinoma

**DOI:** 10.3389/fonc.2021.762629

**Published:** 2022-01-13

**Authors:** Xianhui Liu, Weiyu Zhang, Huanrui Wang, Lin Zhu, Kexin Xu

**Affiliations:** ^1^ Department of Urology, Peking University People’s Hospital, Beijing, China; ^2^ Peking University Applied Lithotripsy Institute, Peking University People’s Hospital, Beijing, China

**Keywords:** renal cancer, ACADSB, prognosis, biomarker, TCGA

## Abstract

**Background:**

Previous reports have shown that short/branched chain acyl-CoA dehydrogenase (ACADSB) plays an important role in glioma, but its role in clear cell renal carcinoma (ccRCC) has not been reported.

**Methods:**

The TIMER and UALCAN databases were used for pan-cancer analysis. RNA sequencing and microarray data of patients with ccRCC were downloaded from the Cancer Genome Atlas and Gene Expression Omnibus database. The differential expression of ACADSB in ccRCC and normal kidney tissues was tested. Correlations between ACADSB expression and clinicopathological parameters were assessed using the Wilcoxon test. The influences of ACADSB expression and clinicopathological parameters on overall survival were assessed using Cox proportional hazards models. Gene set enrichment analysis (GSEA) was performed to explore the associated gene sets enriched in different ACADSB expression phenotypes. Gene ontology (GO) and Kyoto Encyclopedia of Genes and Genomes (KEGG) pathway enrichment analyses were performed on genes with similar expression patterns to ACADSB. Correlations between ACADSB and ferroptosis-related genes were assessed using Spearman’s correlation analysis.

**Results:**

Pan-cancer analysis revealed that ACADSB is down-regulated in multiple cancers, and decreased expression of ACADSB correlates with poor prognosis in certain types of cancer. Differential expression analyses revealed that ACADSB was down-regulated in ccRCC, indicating that ACADSB expression could be a single significant parameter to discriminate between normal and tumor tissues. Clinical association analysis indicated that decreased ACADSB expression was associated with high tumor stage and grade. The Cox regression model indicated that low ACADSB expression was an independent risk factor for the overall survival of patients with ccRCC. GSEA showed that 10 gene sets, including fatty acid (FA) metabolism, were differentially enriched in the ACADSB high expression phenotype. GO and KEGG pathway enrichment analysis revealed that ACADSB-related genes were significantly enriched in categories related to FA metabolism, branched-chain amino acid (BCAA) metabolism, and iron regulation. Spearman’s correlation analysis suggested that the expression of ACADSB was positively correlated with the expression of ferroptosis driver genes.

**Conclusions:**

ACADSB showed good diagnostic and prognostic abilities for ccRCC. The downregulation of ACADSB might promote tumorigenesis and tumor progression by inhibiting FA catabolism, BCAA catabolism, and ferroptosis in ccRCC.

## Introduction

Short/branched chain acyl-CoA dehydrogenase (ACADSB) is a member of the acyl-CoA dehydrogenase family of enzymes, which is involved in the metabolism of fatty acids (FAs) and branch-chained amino acids (BCAAs) (1). Previous studies have revealed that ACADSB plays an important role in glioma, colorectal cancer (CRC), and hepatocellular carcinoma (HCC) (2–5). However, the role of ACADSB in clear cell renal cell carcinoma (ccRCC) has not yet been reported.

CcRCC is the most common and aggressive type of renal cell carcinoma (RCC), which accounts for approximately 3% of all cancers and is the third most common malignancy of the urinary system (6, 7). Worldwide, there were an estimated 403,000 new cases of RCC and 175,000 deaths due to kidney cancer in 2020 (8). Among solid tumors, ccRCC is one of the most resistant to conventional chemotherapy and radiotherapy. Targeted therapy and immune checkpoint inhibitor-based immunotherapy have substantially improved the outcomes of patients with advanced ccRCC over the past decade. However, the identification of novel diagnostic markers and therapeutic targets remains a high priority. The aim of this study was to explore the potential diagnostic and prognostic roles of ACADSB in ccRCC.

## Materials and Methods

### Pan-Cancer Analysis

The tumor immune estimation resource database (TIMER; https://cistrome.shinyapps.io/timer/) (9) and the UALCAN database (http://ualcan.path.uab.edu/index.html) (10) were used for the differential expression analysis of ACADSB between tumor tissues and corresponding normal tissues. TIMER was also applied to explore the association between ACADSB expression and overall survival (OS) in different types of cancers.

### Collection of ccRCC Datasets

The RNA sequencing (RNA-seq) data and clinical information of patients with ccRCC (TCGA-KIRC) were downloaded from The Cancer Genome Atlas (TCGA) using the “TCGAbiolinks” R package (11). Only patients with both RNA-seq data and valid clinical information were included in this study, and duplicated samples were excluded. The expression profiling microarray data of GSE36895 (with 29 ccRCC samples and 23 adjacent normal samples) and GSE53757 (with 72 paired ccRCC and adjacent normal samples) were downloaded from Gene Expression Omnibus (GEO) using the “GEOquery” R package (12).

### Immunohistochemistry (IHC)

A tissue chip with 90 pairs of ccRCC and corresponding normal tissues was purchased from Outdo Biotech Co., Ltd. (HKidE180Su03, Shanghai, China). The experiment received ethical approval for sample use from Shanghai Outdo Biotech Co., Ltd. (Barcode: YB M‐05‐02). IHC was performed on tissues fixed with formaldehyde and embedded in paraffin wax. After deparaffinization and rehydration, the endogenous peroxidase activity was blocked and antigen retrieval was performed. The ACADSB antibody (13122‐1‐AP, 1:12000; Proteintech, CA) was incubated overnight at 4°C. After careful washing and incubation with the specified horseradish peroxidase (HRP)‐conjugated secondary antibody, ACADSB expression was detected using 3,3N‐ diaminobenzidine tetrahydrochloride (DAB).

The intensity and extent of ACADSB staining were evaluated by two experienced pathologists. The method for calculating the score of ACADSB staining was as follows: the extent of staining in an ×200 field was scored as 0, 0%; 1, 1–25%; 2, 26–50%; and 3, 51–100%. The intensity of staining was scored as 0, no signal; 1, light brown; 2, brown; and 3, dark brown. The final score of each field was the average obtained from the two pathologists by multiplying the extent score by the intent score. The scores of ACADSB staining were categorized as follows: low expression (−/+) for scores 0–1 (−) and 2–3 (+) and high expression (++/+++) for scores 4–6 (++) and 7–9 (+++). All evaluations were performed using a Leica DM4000 M microscope.

### Gene Set Enrichment Analysis

Gene-set enrichment analysis (GSEA) (13) was performed with the GSEA software (version 4.0.3). Samples from TCGA were divided into two groups based on the expression of ACADSB. The Broad Molecular Signatures Database (MSigDB v6.0) (14) set H (hallmark gene sets, 50 gene sets) were used, which summarize and represent specific well-defined biological states and pathway processes. Enrichment analysis was performed by default weighted enrichment statistics, with the random combinatorial count set as 1,000. Gene sets were judged as significantly enriched by P<0.05 as well as false discovery rates (FDR) < 0.25.

### Correlation, GO and KEGG Pathway Analysis

The genes which had greater than 0.4 Spearman correlation coefficient with ACADSB in expression level were defined as ACADSB-related genes. To explore the functional annotation and involved pathways of ACADSB-related genes, the gene ontology (GO) and Kyoto Encyclopedia of Genes and Genomes (KEGG) pathway enrichment analyses were executed *via* the “clusterprofiler” package (15).

### Statistical Analysis

All statistical analyses were performed in R version 4.1.0. For RNA-seq data, raw counts data were used for differential expression analysis *via* the “DESeq2” package (16). Fragments per kilobase million (FPKM) values were converted to Transcripts per million (TPM) and log2 transformed for further analysis. For microarray data, differential expression analysis was performed using the “limma” package (17). Genes with an adjusted p value of less than 0.05 and fold change (FC) greater than 2 were regarded as differentially expressed genes (DEGs). The receiver operating characteristic (ROC) curves were plotted and areas under the curve (AUC) were calculated to investigate the diagnostic performance of ACADSB. Patients were stratified into low or high groups based on ACADSB expression, using the median expression as the cut-off value. The relationships between clinical pathologic characteristics and ACADSB expression were analyzed with Chi-squared test or Wilcoxon test. Univariate and multivariate Cox proportional hazards models were used to compare the influence of ACADSB expression on OS along with other clinicopathological parameters. P< 0.05 was considered to indicate a statistically significant difference.

## Results

### ACADSB Is Down-Regulated in Multiple Cancers and Decreased Expression of ACADSB Correlates With Poor Prognosis in Certain Types of Cancers

**Figure 1:**
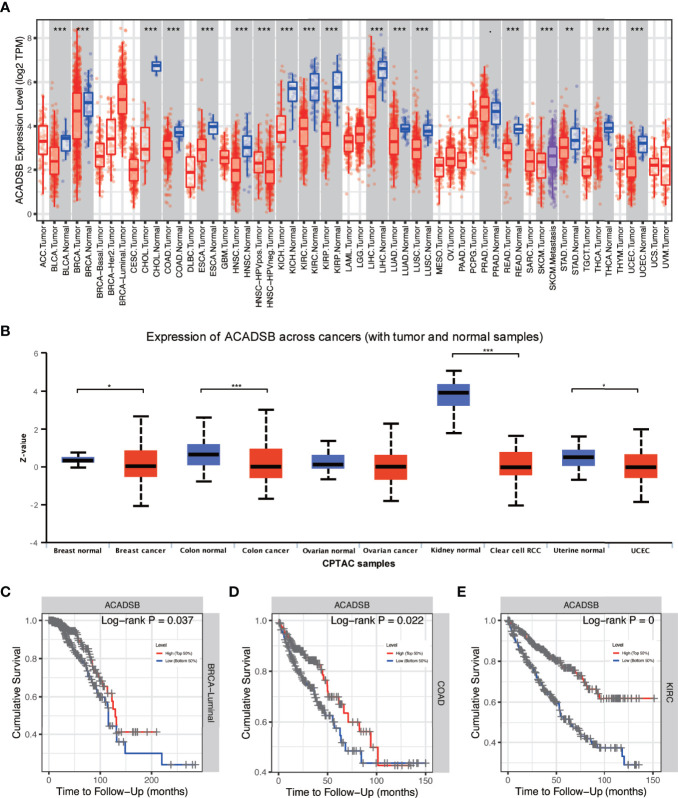
Pan-cancer analysis of ACADSB. **(A)** Differential expressions of ACADSB in TCGA. **(B)** Differential expressions of ACADSB in UALCAN. Overall survivals comparison between high and low ACADSB groups in **(C)** BRCA-luminal subtype, **(D)** COAD, and **(E)** KIRC *p< 0.05; **p<0.01; ***p<0.001.

Differential expression analysis using TIMER showed that ACADSB is down-regulated in multiple types of cancer, including bladder cancer (BLCA), breast cancer (BRCA), cholangiocarcinoma (CHOL), colon adenocarcinoma (COAD), esophageal carcinoma (ESCA), head and neck squamous cell carcinoma (HNSC), kidney chromophobe (KICH), kidney renal clear cell carcinoma (KIRC), kidney renal papillary cell carcinoma (KIRP), liver hepatocellular carcinoma (LIHC), lung adenocarcinoma (LUAD), lung squamous cell carcinoma (LUSC), rectum adenocarcinoma (READ), stomach adenocarcinoma (STAD), thyroid carcinoma (THCA), and uterine corpus endometrial carcinoma (UCEC; **Figure 1A**). Proteome data derived from the UALCAN database also suggest that ACADSB is down-regulated in breast cancer, colon cancer, ccRCC, and UCEC (**Figure 1B**). In addition, Kaplan–Meier analysis indicated that low expression of ACADSB is associated with poor prognosis in BRCA luminal subtype, COAD, KIRC, brain lower grade glioma (LGG), and mesothelioma (MESO) (**Figures 1C–E** and **Supplementary Figures 1A, B**).

### ACADSB Is Down-Regulated in Different ccRCC Datasets

**Figure 2:**
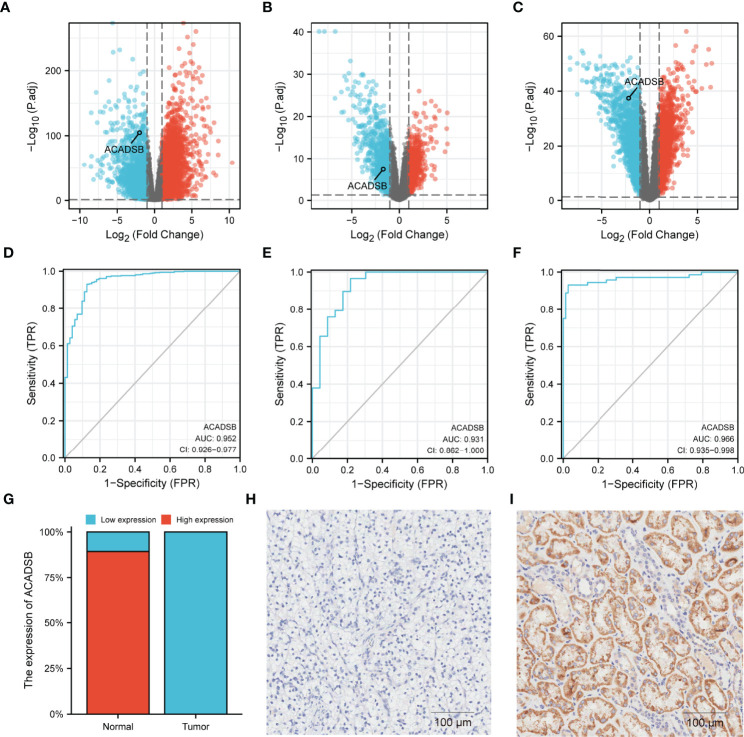
Differential expressions of ACADSB in ccRCC datasets. Volcano plots showing the results of differential analyses in **(A)** TCGA-KIRC, **(B)** GSE36895, and **(C)** GSE53757. ROC curves showing the ability of ACADSB expression to discriminate between normal and tumor tissues in **(D)** TCGA-KIRC, **(E)** GSE36895, and **(F)** GSE53757. **(G)** The IHC result of ccRCC tissue microarray. Typical pictures of paired **(H)** tumor and **(I)** normal tissue.

Transcript differential expression analysis using DESeq2 revealed that ACADSB was significantly down-regulated in ccRCC tissues compared with normal kidney tissues in TCGA-KIRC (log2FC = -2.0, FDR < 0.001; **Figure 2A**), GSE36895 (log2FC = -1.7, FDR < 0.001; **Figure 2B**), and GSE53757 (log2FC = -2.2, FDR < 0.001; **Figure 2C**). Further, ROC analyses showed that ACADSB expression could be a single significant parameter for discriminating between normal and tumor tissues in TCGA-KIRC (AUC = 0.952, 95% CI = 0.926–0.977, **Figure 2D**), GSE36895 (AUC = 0.931, 95% CI = 0.862–1.0, **Figure 2E**), and GSE53757 (AUC = 0.966, 95% CI = 0.935–0.998, **Figure 2F**). A total of 84 paired tumor–normal samples were included in the IHC analysis, with six pairs of samples excluded due to slices escaping from the glass slide. For normal kidney tissues, most samples (75/84, 89.3%) showed high ACADSB expression, while all ccRCC samples (84/84, 100%) showed low ACADSB expression (**Figure 2G**). Typical pictures of staining in paired tumor and normal samples are shown in **Figures 2H, I**.

### Clinical Characteristics of Patients With ccRCC in the TCGA-KIRC Cohort

In the TCGA-KIRC cohort, there’re 530 cases with both RNA-seq data and clinical information. Four duplicated cases and 4 cases whose overall survival time was 0 days were excluded. Therefore, a total of 522 patients with both RNA-seq data and valid survival information were included in this study. The detailed clinical characteristics are listed in **Table 1**. Patients were divided into a high expression group and a low expression group based on ACADSB expression. Among the 522 participants, 341 (65.3%) were men and 181 (34.7%) were women, with a median age of 61 years at the time of initial diagnosis; 315 (60.7%) patients had stage I or stage II disease pathology, and 204 (39.3%) patients had stage III or stage IV disease pathology. Regarding histological grade, 236 (45.9%) patients were categorized into grade 1 or grade 2, and 278 (54.1%) patients were categorized into grade 3 or grade 4. Follow-up duration was 40 months on average (range, 0.1–151.2 months). At the time of the last follow-up, 344 (68.9%) patients were tumor-free, while 155 (31.1%) patients still had tumors.

**Table 1:** Clinical characteristics of patients with ccRCC in TCGA.

Characteristic	Low expression	High expression	p
N	261	261	
Sex, n (%)			0.066
Female	80 (15.3%)	101 (19.3%)	
Male	181 (34.7%)	160 (30.7%)	
Pathological T stage, n (%)			< 0.001
T1-2	138 (26.4%)	195 (37.4%)	
T3-4	123 (23.6%)	66 (12.6%)	
Pathological N stage, n (%)			0.057
N0	112 (44.1%)	126 (49.6%)	
N1	12 (4.7%)	4 (1.6%)	
Pathological M stage, n (%)			< 0.001
M0	188 (38.2%)	226 (45.9%)	
M1	53 (10.8%)	25 (5.1%)	
Pathological stage, n (%)			< 0.001
Stage I-II	125 (24.1%)	190 (36.6%)	
Stage III-IV	133 (25.6%)	71 (13.7%)	
Histological grade, n (%)			< 0.001
G1-2	91 (17.7%)	145 (28.2%)	
G3-4	169 (32.9%)	109 (21.2%)	
Tumor status, n (%)			< 0.001
Tumor free	138 (27.7%)	206 (41.3%)	
With tumor	112 (22.4%)	43 (8.6%)	
Age, median (IQR)	61 (53, 70)	60 (51, 69)	0.423

ccRCC, clear cell renal cell carcinoma; TCGA, The Cancer Genome Atlas; IQR, interquartile range.

### Association Between ACADSB Expression and Clinicopathological Characteristics

**Figure 3:**
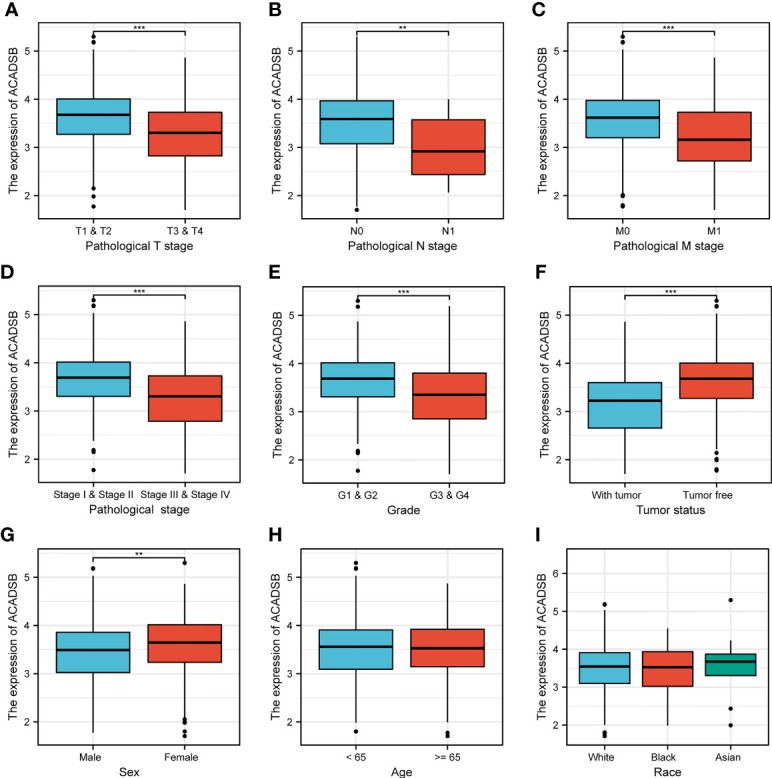
The correlation between ACADSB expression and clinicopathological characteristics. **(A)** Pathological T stage. **(B)** Pathological N stage. **(C)** Pathological M stage. **(D)** Pathological stage. **(E)** Tumor grade. **(F)** Tumor status. **(G)** Sex. **(H)** Age. **(I)** Race *p< 0.05; **p<0.01; ***p<0.001.

The relationship between ACADSB expression and clinicopathological characteristics is shown in **Figure 3**. ACADSB expression was significantly associated with pathological T stage (T1-2 vs. T3-4, P< 0.001; **Figure 3A**), pathological N stage (N0 vs. N1, P< 0.01; **Figure 3B**), pathological M stage (M0 vs. M1, P< 0.001; **Figure 3C**), pathological stage (stage I-II vs. stage III-IV, P< 0.001; **Figure 3D**), tumor grade (grades 1–2 vs. grades 3–4, P< 0.001; **Figure 3E**), tumor status (tumor free vs. with tumor, P< 0.001; **Figure 3F**), and sex (P< 0.01; **Figure 3G**). The differences between groups stratified by age (<65 years vs. ≥65 years; **Figure 3H**) and race (white vs. black or African American vs. Asian; **Figure 3I**) did not attain statistical significance.

### Survival Outcomes and Cox Regression Analysis

**Figure 4:**
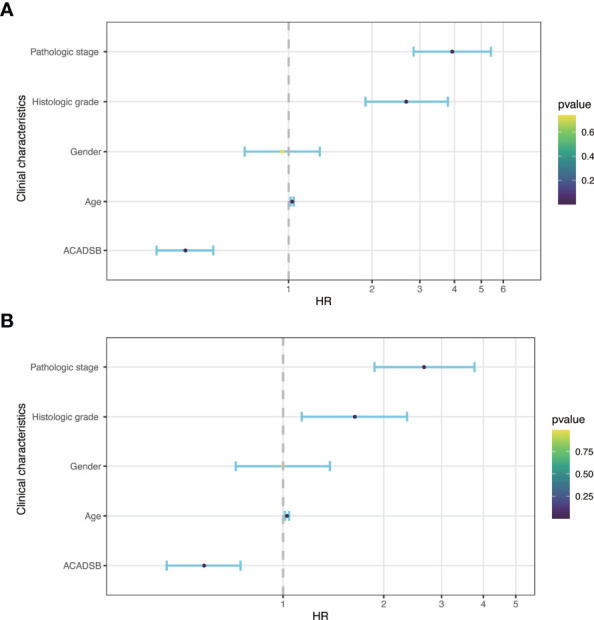
Forest plot of hazard ratios derived from Cox model. **(A)** Univariate analysis. **(B)** Multivariate analysis.

Kaplan–Meier survival analysis showed that lower ACADSB expression was associated with a worse prognosis (P< 0.001; **Figure 1E**). The univariate Cox regression model revealed that age at diagnosis (HR = 1.03, 95% CI = 1.017–1.044, P< 0.001), pathological stage (HR = 3.927, 95% CI = 2.847–5.417, P< 0.001), histological grade (HR = 2.679, 95% CI = 1.895–3.787, P< 0.001), and ACADSB expression (HR = 0.421, 95% CI = 0.331–0.535, P< 0.001) were associated with the OS of patients with ccRCC. Multivariate Cox regression after adjustment indicated that age at diagnosis (HR = 1.027, 95% CI = 1.013–1.042, P< 0.001), pathological stage (HR = 2.695, 95% CI = 1.877–3.765, P< 0.001), histological grade (HR = 1.639, 95% CI = 1.137–2.364, P< 0.01), and ACADSB expression (HR = 0.577, 95% CI = 0.446–0.746, P< 0.001) were independent prognostic factors for OS in patients with ccRCC (**Figure 4** and **Supplementary Table 1**).

### Gene Sets Enriched in the ACADSB High Expression Phenotype

**Figure 5:**
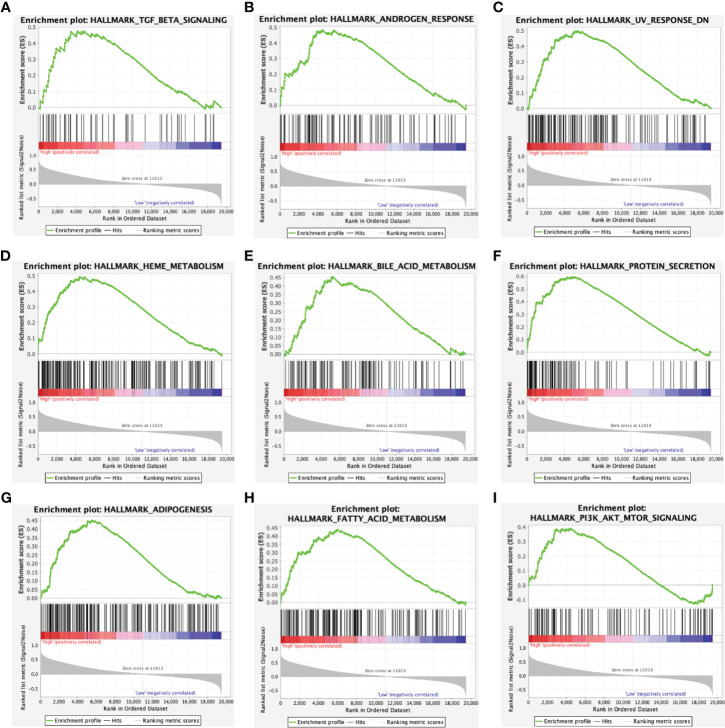
Enrichment plots from gene set enrichment analysis. Gene sets enriched in ACADSB high phenotype: **(A)** TGF-β signaling. **(B)** Androgen response. **(C)** UV response down. **(D)** Heme metabolism. **(E)** Bile acid metabolism. **(F)** Protein secretion. **(G)** Adipogenesis. **(H)** Fatty acid metabolism. **(I)** PI3K-AKT-mTOR signaling.

In the hallmark dataset, 10 gene sets were significantly enriched in the ACADSB high expression phenotype, including TGF-β signaling, androgen response, UV response down, heme metabolism, bile acid metabolism, protein secretion, adipogenesis, FA metabolism, PI3K-AKT-mTOR signaling, and mitotic spindle (**Figure 5** and **Supplementary Figure 1C**).

### GO and KEGG Analysis of ACADSB-Related Genes

**Figure 6:**
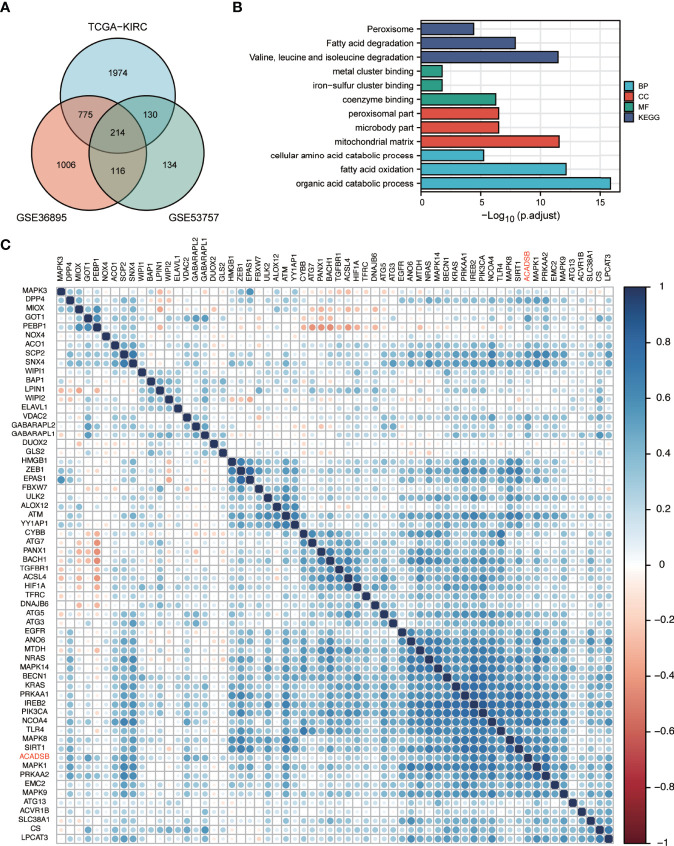
GO and KEGG enrichment analyses of ACADSB-related genes and correlation between ACADSB with ferroptosis-related genes. **(A)** Identification of ACADSB-related genes. **(B)** GO and KEGG enrichment analyses of ACADSB-related genes. **(C)** Heatmap shows the correlation between ACADSB and ferroptosis driver gens.

In TCGA-KIRC, GSE36895, and GSE53757, 3093, 2111, and 594 respective genes had ACADSB expression with a Spearman correlation coefficient of >0.4. Two hundred and fourteen genes representing the intersection between all three datasets were defined as ACADSB-related genes (**Figure 6A**). At a false discovery rate (FDR) of 0.05, 47 GO biological processes, 13 GO cellular components, 14 GO molecular functions, and 15 KEGG pathways were significantly enriched for these genes (**Figure 6B** and **Supplementary Table 2**).

### ACADSB Expression Was Associated With the Expression of Ferroptosis-Related Genes

Ferroptosis-related genes were derived from the FerrDb database (18). A total of 108 genes were identified as ferroptosis driver genes. Correlation analysis revealed that the expression of ACADSB was significantly positively correlated with the expression of 62 ferroptosis diver genes in the TCGA-KIRC dataset (**Figure 6C**). Similar results were found in GSE36895 and GSE53757 (**Supplementary Figure 1D, E**).

## Discussion

ACADSB is a member of the acyl-CoA dehydrogenase family of enzymes that catalyze the dehydrogenation of acyl-CoA derivatives in the metabolism of FAs and BCAAs (1). ACADSB is widely known to be associated with ACADSB deficiency (SBCADD), an autosomal recessive disorder characterized by seizures and psychomotor delay due to a defect in the catabolism of L-isoleucine (19, 20).

Recent studies have found that ACADSB plays an important role in the development and progression of malignant diseases, such as glioma (2), colorectal cancer (3), and hepatocellular carcinoma (HCC) (4, 5). Yu et al. (2) reported that ACADSB is lowly expressed in high-grade gliomas, and the lower expression of ACADSB may lead to the accumulation of short-chain acylcarnitines, which further facilitates the growth and progression of gliomas. Di et al. (3) reported that ACADSB is down-regulated in CRC, and its expression is positively correlated with the OS of patients with CRC. Their study also indicated that overexpression of ACADSB inhibits CRC cell proliferation, migration, and invasion, while knockdown of ACADSB has the opposite effect. Similar results were also found in HCC, in which the restoration of ACADSB expression caused mTORC1 activity and cell proliferation to significantly decrease (4). In this study, we first explored the expression levels of ACADSB in different types of cancer using independent datasets from TCGA and UALCAN. The downregulation of ACADSB was observed in almost all types of common cancer. Moreover, low expression levels of ACADSB also correlate with a poorer prognosis in many types of cancers, such as COAD, LGG, and ccRCC. Previous studies have reported the important role of ACADSB in COAD and LGG; however, it has not been reported in ccRCC. Thus, we focused on the role of ACADSB in ccRCC.

The differential expression analyses were performed in three independent datasets, and consistent results were obtained, indicating that ACADSB is down-regulated in ccRCC, and that ACADSB expression can be a single significant parameter to discriminate between normal and tumor tissues. The IHC results further validated the downregulation of ACADSB in ccRCC. We next investigated the correlation between ACADSB expression and the clinicopathological characteristics of patients with ccRCC using TCGA-KIRC data. Results showed that the level of ACADSB expression was negatively correlated with tumor stage and tumor grade; results also showed that ACADSB expression was an independent prognostic factor for OS independent of conventional prognostic factors, such as age at diagnosis, tumor stage, and tumor grade. These findings indicate an integral role of ACADSB in the underlying biological mechanisms of tumor development and progression of ccRCC.

To further investigate the potential functions of ACADSB in ccRCC, we conducted GSEA, GO, and KEGG analyses. The results of GSEA showed that 10 gene sets in the hallmark dataset, including FA metabolism, bile acid metabolism, heme metabolism, and adipogenesis, were differentially enriched in the ACADSB high expression phenotype. GO and KEGG enrichment analysis indicated that ACADSB-related genes were enriched in the regulation of FA degeneration and BCAA degeneration. Lip metabolism plays a critical role in the development and progression of renal cancer, and ccRCC is histologically defined by its lipid-and glycogen-rich cytoplasmic deposits (21). The tumorigenic role of lipid accumulation has been observed in many types of cancer (22, 23). ACADSB is known to regulate FA catabolism by catalyzing the dehydrogenation of acyl-CoA derivatives (1). Thus, the downregulation of ACADSB may contribute to tumor development by suppressing FA catabolism, resulting in lipid accumulation in ccRCC.

In addition to regulating FA catabolism, ACADSB also catalyzes the dehydrogenation of acyl-CoA derivatives in the metabolism of BCAAs. BCAA metabolism can influence multiple cancer phenotypes and serve as a marker of disease pathology (24). Previous studies have demonstrated that BCAA catabolism is reduced in many types of cancers, such as HCC (4), ccRCC (22), and breast cancer (25). Ericksen et al. (4) found that suppression of BCAA catabolic enzyme expression led to BCAA accumulation in liver tumors and that progressive loss of BCAA catabolism promoted tumor development and growth. Qu et al. (22) reported that BCAA accumulation in ccRCC induced the activation of mTORC1 and *de novo* FA synthesis and promoted cell proliferation. Therefore, the downregulation of ACADSB may also promote tumor development and growth by inhibiting BCAA catabolism.

Lipid metabolism is important for ferroptosis, an iron-dependent form of regulated cell death (RCD) that is driven by the lethal accumulation of lipid peroxidation (26, 27) and plays a key role in tumor suppression (28). In contrast, the glutathione peroxidase 4 (GPX4)/glutathione (GSH) antioxidation system acts as an endogenous antioxidant pathway to suppress ferroptosis (29, 30). Reduced FA metabolism due to inhibition of β-oxidation renders renal cancer cells highly dependent on the GPX4/GSH pathway to prevent lipid peroxidation and ferroptosis (31). Lu et al. (3) found that overexpression of ACADSB enhanced the concentrations of Fe+ and lipid peroxidation but reduced the concentration of GSH and the expression of GPX4 in CRC cell lines, suggesting a potential regulatory effect of ACADSB on CRC cell ferroptosis. In this study, GSEA indicated that FA metabolism and heme metabolism were enriched in the high-ACADSB phenotype, GO analysis revealed that ACADSB-related genes are enriched in the molecular function of metal cluster binding and iron-sulfur cluster binding, and KEGG analysis revealed that ACADSB-related genes are enriched in the peroxisome pathway. Taken together, these findings indicate the potential regulatory effect of ACADSB on ferroptosis in ccRCC.

Ferroptosis-related genes can be classified as either ferroptosis driver genes that promote ferroptosis or ferroptosis suppressor genes that prevent ferroptosis (18). Correlation analysis revealed that the expression of ACADSB was significantly positively correlated with the expression of ferroptosis driver genes, including PRKAA1, PRKAA2, and NCOA4. Song et al. (32) demonstrated that the inhibition of PRKAA1/AMPKα1 or PRKAA2/AMPKα2 by siRNA diminished erastin-induced BECN1 phosphorylation at S93/96, BECN1-SLC7A11 complex formation, and subsequent ferroptosis. Hou et al. (33) revealed that the genetic inhibition of NCOA4 inhibited ferritin degradation and suppressed ferroptosis. Taken together, ACADSB might also be a potential ferroptosis driver gene, and the downregulation of ACADSB in ccRCC may promote tumor progression by suppressing ferroptosis.

In conclusion, ACADSB is down-regulated in multiple types of cancers and shows good diagnostic and prognostic abilities in ccRCC. Bioinformatic analyses revealed that ACADSB might affect the development and progression of ccRCC by regulating FA catabolism, BCAA catabolism, and ferroptosis. These findings may offer new therapeutic approaches for the clinical treatment and prognostic assessment of ccRCC. However, there were some limitations to this study. This study was mainly conducted using data from the public databases, and the potential regulations between ACADSB and ferroptosis in ccRCC were analyzed by correlation analysis, which needs to be further elucidated by molecular experiments.

## Data Availability Statement

Publicly available datasets were analyzed in this study. This data can be found here: https://portal.gdc.cancer.gov/, https://www.ncbi.nlm.nih.gov/geo/query/acc.cgi?acc=GSE36895, https://www.ncbi.nlm.nih.gov/geo/query/acc.cgi?acc=GSE53757.

## Ethics Statement

The studies involving human participants were reviewed and approved by Ethics Committee of Shanghai Outdo Biotech Company Limited. Written informed consent for participation was not required for this study in accordance with the national legislation and the institutional requirements.

## Author Contributions

KX and XL conceived the manuscript. XL and WZ designed and wrote the manuscript. HW and LZ helped with the bioinformatic analyses. All authors contributed to the article and approved the submitted version.

## Funding

The present study was supported by the National Natural Science Foundation of China (NO. 81970660) from KX.

## Conflict of Interest

The authors declare that the research was conducted in the absence of any commercial or financial relationships that could be construed as a potential conflict of interest.

## Publisher’s Note

All claims expressed in this article are solely those of the authors and do not necessarily represent those of their affiliated organizations, or those of the publisher, the editors and the reviewers. Any product that may be evaluated in this article, or claim that may be made by its manufacturer, is not guaranteed or endorsed by the publisher.

## References

[1] Rozen R Vockley J Zhou L Milos R Willard J Fu K. Isolation and Expression of a cDNA Encoding the Precursor for a Novel Member (ACADSB) of the Acyl-CoA Dehydrogenase Gene Family. Genomics (1994) 24(2):280–7. doi: 10.1006/geno.1994.1617 7698750

[2] Yu D Xuan Q Zhang C Hu C Li Y Zhao X. Metabolic Alterations Related to Glioma Grading Based on Metabolomics and Lipidomics Analyses. Metabolites (2020) 10(12):478. doi: 10.3390/metabo10120478 PMC7760389 33255474

[3] Lu D Yang Z Xia Q Gao S Sun S Luo X. ACADSB Regulates Ferroptosis and Affects the Migration, Invasion, and Proliferation of Colorectal Cancer Cells. Cell Biol Int (2020) 44(11):2334–43. doi: 10.1002/cbin.11443 32776663

[4] Ericksen RE Lim SL McDonnell E Shuen WH Vadiveloo M White PJ. Loss of BCAA Catabolism During Carcinogenesis Enhances Mtorc1 Activity and Promotes Tumor Development and Progression. Cell Metab (2019) 29(5):1151–65.e6. doi: 10.1016/j.cmet.2018.12.020 PMC6506390 30661928

[5] Sun R Xu Y Zhang H Yang Q Wang K Shi Y. Mechanistic Modeling of Gene Regulation and Metabolism Identifies Potential Targets for Hepatocellular Carcinoma. Front Genet (2020) 11:595242. doi: 10.3389/fgene.2020.595242 33424926 PMC7786279

[6] Capitanio U Bensalah K Bex A Boorjian SA Bray F Coleman J. Epidemiology of Renal Cell Carcinoma. Eur Urol (2019) 75(1):74–84. doi: 10.1016/j.eururo.2018.08.036 30243799 PMC8397918

[7] Moch H Cubilla AL Humphrey PA Reuter VE Ulbright TM. The 2016 WHO Classification of Tumours of the Urinary System and Male Genital Organs-Part A: Renal, Penile, and Testicular Tumours. Eur Urol (2016) 70(1):93–105. doi: 10.1016/j.eururo.2016.02.029 26935559

[8] Siegel RL Miller KD Jemal A. Cancer Statistics, 2020. CA Cancer J Clin (2020) 70(1):7–30. doi: 10.3322/caac.21590 31912902

[9] Li T Fan J Wang B Traugh N Chen Q Liu JS. TIMER: A Web Server for Comprehensive Analysis of Tumor-Infiltrating Immune Cells. Cancer Res (2017) 77(21):e108–e10. doi: 10.1158/0008-5472.CAN-17-0307 PMC6042652 29092952

[10] Chandrashekar DS Bashel B Balasubramanya SAH Creighton CJ Ponce-Rodriguez I Chakravarthi BVSK. UALCAN: A Portal for Facilitating Tumor Subgroup Gene Expression and Survival Analyses. Neoplasia (2017) 19(8):649–58. doi: 10.1016/j.neo.2017.05.002 PMC5516091 28732212

[11] Colaprico A Silva TC Olsen C Garofano L Cava C Garolini D. TCGAbiolinks: An R/Bioconductor Package for Integrative Analysis of TCGA Data. Nucleic Acids Res (2016) 44(8):e71. doi: 10.1093/nar/gkv1507 26704973 PMC4856967

[12] Davis S Meltzer PS. GEOquery: A Bridge Between the Gene Expression Omnibus (GEO) and BioConductor. Bioinformatics (2007) 23(14):1846–7. doi: 10.1093/bioinformatics/btm254 17496320

[13] Subramanian A Tamayo P Mootha VK Mukherjee S Ebert BL Gillette MA. Gene Set Enrichment Analysis: A Knowledge-Based Approach for Interpreting Genome-Wide Expression Profiles. Proc Natl Acad Sci USA (2005) 102(43):15545–50. doi: 10.1073/pnas.0506580102 PMC1239896 16199517

[14] Liberzon A Birger C Thorvaldsdottir H Ghandi M Mesirov JP Tamayo P. The Molecular Signatures Database (MSigDB) Hallmark Gene Set Collection. Cell Syst (2015) 1(6):417–25. doi: 10.1016/j.cels.2015.12.004 PMC4707969 26771021

[15] Yu G Wang L-G Han Y He Q-Y. Clusterprofiler: An R Package for Comparing Biological Themes Among Gene Clusters. OMICS (2012) 16(5):284–7. doi: 10.1089/omi.2011.0118 PMC3339379 22455463

[16] Love MI Huber W Anders S. Moderated Estimation of Fold Change and Dispersion for RNA-Seq Data With Deseq2. Genome Biol (2014) 15(12):550. doi: 10.1186/s13059-014-0550-8 25516281 PMC4302049

[17] Ritchie ME Phipson B Wu D Hu Y Law CW Shi W. Limma Powers Differential Expression Analyses for RNA-Sequencing and Microarray Studies. Nucleic Acids Res (2015) 43(7):e47. doi: 10.1093/nar/gkv007 25605792 PMC4402510

[18] Zhou N Bao J. FerrDb: A Manually Curated Resource for Regulators and Markers of Ferroptosis and Ferroptosis-Disease Associations. Database (Oxf) (2020) 2020:baaa021. doi: 10.1093/database/baaa021 PMC7100629 32219413

[19] Gibson KM Burlingame TG Hogema B Jakobs C Schutgens RB Millington D. 2-Methylbutyryl-Coenzyme A Dehydrogenase Deficiency: A New Inborn Error of L-Isoleucine Metabolism. Pediatr Res (2000) 47(6):830–3. doi: 10.1203/00006450-200006000-00025 10832746

[20] Andresen BS Christensen E Corydon TJ Bross P Pilgaard B Wanders RJ. Isolated 2-Methylbutyrylglycinuria Caused by Short/Branched-Chain Acyl-CoA Dehydrogenase Deficiency: Identification of a New Enzyme Defect, Resolution of Its Molecular Basis, and Evidence for Distinct Acyl-CoA Dehydrogenases in Isoleucine and Valine Metabolism. Am J Hum Genet (2000) 67(5):1095–103. doi: 10.1086/303105 PMC1288551 11013134

[21] Du W Zhang L Brett-Morris A Aguila B Kerner J Hoppel CL. HIF Drives Lipid Deposition and Cancer in ccRCC *via* Repression of Fatty Acid Metabolism. Nat Commun (2017) 8(1):1769. doi: 10.1038/s41467-017-01965-8 29176561 PMC5701259

[22] Qu Y-Y Zhao R Zhang H-L Zhou Q Xu F-J Zhang X. Inactivation of the AMPK-GATA3-ECHS1 Pathway Induces Fatty Acid Synthesis That Promotes Clear Cell Renal Cell Carcinoma Growth. Cancer Res (2020) 80(2):319–33. doi: 10.1158/0008-5472.CAN-19-1023 31690668

[23] Carracedo A Cantley LC Pandolfi PP. Cancer Metabolism: Fatty Acid Oxidation in the Limelight. Nat Rev Cancer (2013) 13(4):227–32. doi: 10.1038/nrc3483 PMC3766957 23446547

[24] Sivanand S Vander Heiden MG. Emerging Roles for Branched-Chain Amino Acid Metabolism in Cancer. Cancer Cell (2020) 37(2):147–56. doi: 10.1016/j.ccell.2019.12.011 PMC7082774 32049045

[25] Zhang L Han J. Branched-Chain Amino Acid Transaminase 1 (BCAT1) Promotes the Growth of Breast Cancer Cells Through Improving mTOR-Mediated Mitochondrial Biogenesis and Function. Biochem Biophys Res Commun (2017) 486(2):224–31. doi: 10.1016/j.bbrc.2017.02.101 28235484

[26] Dixon SJ Lemberg KM Lamprecht MR Skouta R Zaitsev EM Gleason CE. Ferroptosis: An Iron-Dependent Form of Nonapoptotic Cell Death. Cell (2012) 149(5):1060–72. doi: 10.1016/j.cell.2012.03.042 PMC3367386 22632970

[27] Stockwell BR Friedmann Angeli JP Bayir H Bush AI Conrad M Dixon SJ. Ferroptosis: A Regulated Cell Death Nexus Linking Metabolism, Redox Biology, and Disease. Cell (2017) 171(2):273–85. doi: 10.1016/j.cell.2017.09.021 PMC5685180 28985560

[28] Stockwell BR Jiang X Gu W. Emerging Mechanisms and Disease Relevance of Ferroptosis. Trends Cell Biol (2020) 30(6):478–90. doi: 10.1016/j.tcb.2020.02.009 PMC7230071 32413317

[29] Chen X Kang R Kroemer G Tang D. Broadening Horizons: The Role of Ferroptosis in Cancer. Nat Rev Clin Oncol (2021) 18(5):280–96. doi: 10.1038/s41571-020-00462-0 33514910

[30] Forcina GC Dixon SJ. GPX4 at the Crossroads of Lipid Homeostasis and Ferroptosis. Proteomics (2019) 19(18):e1800311. doi: 10.1002/pmic.201800311 30888116

[31] Miess H Dankworth B Gouw AM Rosenfeldt M Schmitz W Jiang M. The Glutathione Redox System Is Essential to Prevent Ferroptosis Caused by Impaired Lipid Metabolism in Clear Cell Renal Cell Carcinoma. Oncogene (2018) 37(40):5435–50. doi: 10.1038/s41388-018-0315-z PMC6173300 29872221

[32] Song X Zhu S Chen P Hou W Wen Q Liu J. AMPK-Mediated BECN1 Phosphorylation Promotes Ferroptosis by Directly Blocking System X Activity. Curr Biol (2018) 28(15):2388–99.e5. doi: 10.1016/j.cub.2018.05.094 PMC6081251 30057310

[33] Hou W Xie Y Song X Sun X Lotze MT Zeh HJ. Autophagy Promotes Ferroptosis by Degradation of Ferritin. Autophagy (2016) 12(8):1425–8. doi: 10.1080/15548627.2016.1187366 PMC4968231 27245739

