# Supplementary figures and images for: Decreased Expression of ACADSB Predicts Poor Prognosis in Clear Cell Renal Cell Carcinoma
Source: Front Oncol. 2022 Jan 13;11:762629. doi: 10.3389/fonc.2021.762629 (PMC8791850; doi:10.3389/fonc.2021.762629)

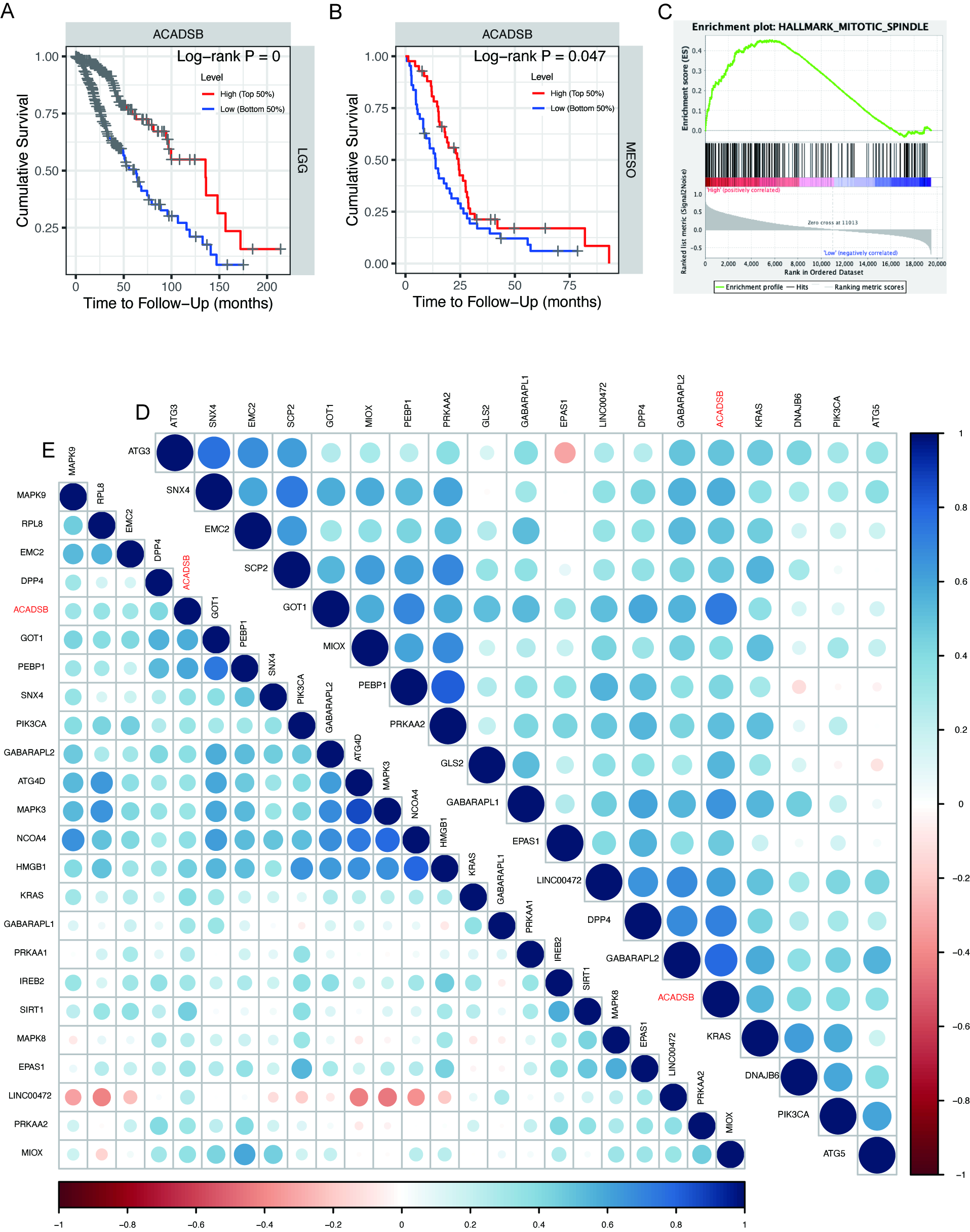

Supplement: Supplementary file 1 [file Image_1.tif]
