# Supplementary material for: Decreased Expression of ACADSB Predicts Poor Prognosis in Clear Cell Renal Cell Carcinoma
Source: Front Oncol. 2022 Jan 13;11:762629. doi: 10.3389/fonc.2021.762629 (PMC8791850; doi:10.3389/fonc.2021.762629)
Supplement: Supplementary file 2 [file Table_1.docx]

Supplementary Table 1. Cox regression analysis of overall survival.

| Characteristics | Univariate analysis | |  | Multivariate analysis | |
| --- | --- | --- | --- | --- | --- |
|  | Hazard ratio (95% CI) | P value |  | Hazard ratio (95% CI) | P value |
| Age | 1.030 (1.017-1.044) | **<0.001** |  | 1.027 (1.013-1.042) | **<0.001** |
| Gender |  |  |  |  |  |
| Femal | Reference |  |  |  |  |
| Male | 0.949 (0.694-1.296) | 0.740 |  |  |  |
| Pathological stage |  |  |  |  |  |
| Stage I-II | Reference |  |  |  |  |
| Stage III-IV | 3.927 (2.847-5.417) | **<0.001** |  | 2.659 (1.877-3.765) | **<0.001** |
| Histological grade |  |  |  |  |  |
| G1-2 | Reference |  |  |  |  |
| G3-4 | 2.679 (1.895-3.787) | **<0.001** |  | 1.639 (1.137-2.364) | **0.008** |
| ACADSB expression | 0.421 (0.331-0.535) | **<0.001** |  | 0.577 (0.446-0.746) | **<0.001** |

HR, hazard ratio; CI, Confidence interval
